# Association between SPARC mRNA Expression, Prognosis and Response to Neoadjuvant Chemotherapy in Early Breast Cancer: A Pooled *in-silico* Analysis

**DOI:** 10.1371/journal.pone.0062451

**Published:** 2013-04-26

**Authors:** Hatem A. Azim, Sandeep Singhal, Michail Ignatiadis, Christine Desmedt, Debora Fumagalli, Isabelle Veys, Denis Larsimont, Martine Piccart, Stefan Michiels, Christos Sotiriou

**Affiliations:** 1 Breast Cancer Translational Research Laboratory, J.C. Heuson, Institut Jules Bordet, Université Libre de Bruxelles, Brussels, Belgium; 2 Department of Medical Oncology, Institut Jules Bordet, Université Libre de Bruxelles, Brussels, Belgium; 3 Department of Breast and Gynecological Surgery, Institut Jules Bordet, Brussels, Belgium; 4 Department of Pathology, Institut Jules Bordet, Brussels, Belgium; Virginia Commonwealth University, United States of America

## Abstract

**Introduction:**

SPARC is an important regulator of the extracellular matrix and has been suggested to improve delivery of albumin-bound cytotoxics. However, little is known regarding its role in breast cancer (BC).

**Methods:**

We conducted a pooled analysis of publically available datasets, in which BC patients who received no systemic therapy or received neoadjuvant chemotherapy were eligible. Patients were assigned to molecular subtypes using PAM-50. We computed a SPARC module (SPARC7), composed of genes with an absolute correlation with SPARC >0.7. In the systemically untreated cohort, we evaluated 1) expression of SPARC/SPARC7 according to breast cancer subtype, 2) association between SPARC/SPARC7 and biological processes related to proliferation, immune and stroma, and 3) association between SPARC/SPARC7 and relapse-free survival in a Cox model in all patients and in the different molecular subtypes adjusted for tumor size, nodal status, histological grade, and age. In the neoadjuvant cohort, we evaluated the association between SPARC and pCR in a logistic regression model, adjusted for the same clinicopathologic factors.

**Results:**

948 (10 datasets), and 791 (8 datasets) patients were included in the systemically untreated and neoadjuvant cohorts, respectively. High SPARC expression was associated with small tumor size, low histological grade and luminal-A tumors (all p<0.0001). There was a positive correlation between SPARC and stroma-related modules but negative correlation with proliferation modules. High SPARC expression was associated with poor prognosis in patients with basal and HER2+ breast cancer even after adjusting for clinicopathologic parameters. In the neoadjuvant cohort, a subgroup analysis suggested that high SPARC is associated with low rates of pCR in the HER2 subtype. Same results were observed on replacing SPARC by SPARC7.

**Conclusion:**

This analysis suggests a potential role of SPARC in determining prognosis and response to primary chemotherapy in early BC. This information could guide further development of albumin-bound cytotoxics in BC.

## Introduction

Breast cancer is the most common malignant tumor in women and the second cause of cancer-related morality [1]. Recent advances in the field of gene expression profiling shed the light on the molecular heterogeneity of this disease [2]. Currently, four main classes of breast cancer have been identified, namely luminal-A, luminal-B, HER2-positive and basal-like [3], [4], [5]. Each has distinct prognosis, and respond differently to systemic therapy [6]. In addition, every subtype has an array of molecular aberrations that derive tumor progression [7].

Among the key factors that govern tumor progression is the cross-talk between the cancer cell and the surrounding microenvironment, which includes stromal and immune cells as well as the extracellular matrix (ECM) [8], [9]. The latter provides a structural framework for the cells and plays a vital role in regulating cell differentiation, migration, proliferation and survival [10]. SPARC (secreted protein acidic and rich in cysteine) emerges as a key regulator of ECM via its interaction with different components like collagen, laminin, vitronectin as well as growth factors like the vascular endothelial growth factor and platelet-derived growth factor [11], [12], [13]. SPARC has been suggested to affect cell cycle progression via regulating the actin-cytoskeleton architecture and integrin-linked kinases [14]. Furthermore, it has been shown to bind with high affinity to albumin, which raised some interest in its possible role in improving the delivery of albumin-bound cytotoxics to the tumor [15], [16].

Limited data are available regarding the potential role of SPARC in breast cancer. Few studies have shown that high SPARC expression is associated with poor prognosis [17], [18], while strikingly others have reported the exact opposite [19]. The small size of the former studies, the use of sub-optimally validated assays to assess SPARC expression and also investigating its association with outcome in unselected population possibly explain these contradictory findings. Hence, we conducted a pooled analysis to evaluate SPARC expression according to BC molecular subtypes and its association with clinical outcome in early BC.

## Methods

### Eligible Datasets

We searched the electronic databases (PubMED, GEO, ArrayExpress) up to July 2012 using the keywords “breast cancer”, “untreated” and after reviewing all retrieved abstracts, we identified studies published in peer-reviewed journals that analyzed gene expression profiling data from patients that did not receive primary (adjuvant or neoadjuvant) systemic therapy for breast cancer. We also used a recently pooled analysis by our group of gene expression profiling data from pretreatment biopsies of patients receiving neoadjuvant anthracycline with or without taxanes (docetaxel, paclitaxel) prior to surgical resection “i.e. neoadjuvant cohort” [20]. The normalized data from all studies has been downloaded from public resources and were used in its original form. No further normalization or transformation was performed.

### Gene Expression Data

Breast cancer molecular subtypes were categorized as basal, HER2, luminal-A and luminal-B based on the PAM-50 classifier [21]. Tumors classified as “normal-like” were excluded. The expression of individual genes was retrieved from public databases (GEO or ArrayExpress or authors website). We computed several gene modules reflecting different biological process; proliferation-related gene modules (AURKA, GGI) [22], [23], immune-related gene modules (immune1 [IRM], immune2 [STAT1]) [23], [24] and stroma-related gene modules (stroma1 [DCN], stroma2 [PLAU]) [23], [25]. Table S1 summarizes the gene list of each module. Thirty-four genes were common between the two proliferation modules, while 13 genes were common between the two stroma-related modules. No genes were in common between the two immune-related modules.

We computed a SPARC-related gene module (named as SPARC7), which is composed of genes with an absolute correlation above 0.7 with SPARC based on data derived from 384 patients in two publically available datasets [26], [27] A module score was defined as:
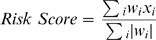
where x_i_ is the expression of a gene in the gene module or gene signatures that is present in the data set platform, and w_i_ is either +1 or −1 depending on the sign of gene-specific statistic from the original studies. Each risk score was scaled such that quantiles 2.5% and 97.5% equaled −1 and +1 respectively to allow for comparison between datasets using different microarray technologies and normalization procedures.

### Statistical Analysis

Within the systemically untreated cohort, pairwise correlations were estimated between the different gene modules using the Pearson correlation. Gene set enrichment analysis (GSEA) was performed using the KEGG Pathways Database (www.genome.jp/kegg/pathway.html) to evaluate the pathways that are enriched according to median SPARC expression (low vs. high). We also evaluated the correlation between SPARC, SPARC7 and clinic-pathological characteristics as well as breast cancer molecular subtypes using Mann-Whitney or Kruskal-Wallis as appropriate.

Relapse-free survival (RFS) was the primary survival endpoint. This was defined as the time elapsing between surgery and date of local or systemic recurrence, or death. The median follow-up was calculated using the reversed Kaplan-Meier method [28]. The systemically untreated cohort was divided into three categories corresponding to the tertile 33% and 66% of SPARC and SPARC7 expression in each dataset. We evaluated the effect of SPARC, SPARC7 in an unadjusted model on RFS using the log-rank test in the systemically untreated cohort. The analysis was performed in the overall patient population, and in subgroups according to the breast cancer molecular subtype. We then evaluated the association between SPARC expression and relapse-free survival in a multivariate model adjusted for tumor size, nodal status, histological grade, estrogen receptor (ER), HER2, age, and stratified for dataset. We tested the interaction between SPARC and ER and HER2. We then conducted a multivariate model within each molecular subtype, which was adjusted for the same clinicopathologic factors. The same analysis was performed replacing SPARC with SPARC7.

To address whether SPARC expression is associated with response to preoperative chemotherapy, we investigated the effect of SPARC, SPARC7 as a continuous variable on pathological complete response (pCR) in the neoadjuvant cohort in a logistic regression model adjusted for the same clinicopathologic parameters. The analyses were performed in all patients, and in subgroups according to breast cancer molecular subtype, all stratified by dataset.

All Reported p-values are two sided. Statistical analyses were performed using R software version 2.15.0 (www.r-project.org).

## Results

The systemically untreated cohort included 948 patients with complete clinical information, which were included from 10 publically available datasets (Table S2). We used the same neoadjuvant dataset that was recently published by our group [20], which included 791 patients. We computed SPARC7, which was composed of 53 genes with absolute correlation >0.7 with SPARC (Table S3).

### Association between SPARC, Breast Cancer Subtypes and Clinicopathologic Characteristics

**Figure 1:**
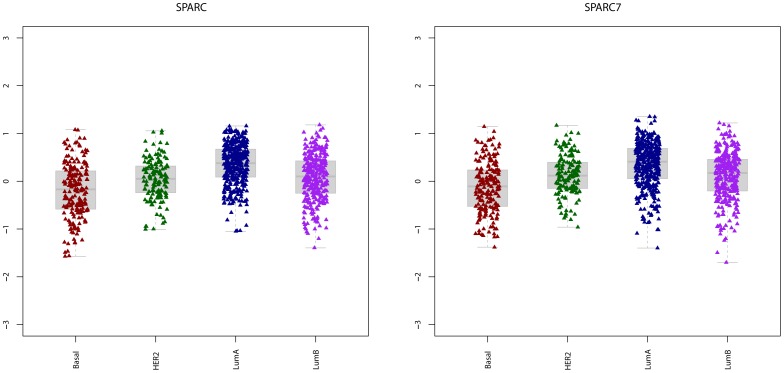
The expression of SPARC & SPARC7 according to breast cancer subtype; basal (n−153), HER2 (n = 67), luminal-A (n = 342), luminal-B (n = 386). **A)** SPARC expression was higher in luminal-A tumors compared to basal (p = 8.31E−27), HER2 (p = 9.42E−13) and luminal-B (p = 9.07E−14). Compared to basal, SPARC expression was higher in HER2 (p = 1.13E−05) and luminal-B (p = 6.77E−08). No difference in SPARC expression was observed between luminal-B and HER2 (p = 4.39E−01). **B)** SPARC7 expression was higher in luminal-A tumors compared to basal (p = 2.29E−21), HER2 (p = 1.57E−06) and luminal-B (p = 1.23E−08). Compared to basal, SPARC expression was higher in HER2 (p = 2.86E−06) and luminal-B (p = 1.34E−06). No difference in SPARC expression was observed between luminal-B and HER2 (p = 8.12E−01).

In the systemically untreated cohort (n = 948), we evaluated the association between SPARC/SPRAC7expression, breast cancer subtypes (Figure 1) and clinicopathologic parameters (Table 1). We observed a significantly higher expression of SPARC and SPARC7 in luminal-A (Figure 1) compared to any of the other subtypes. SPARC and SPARC7 were significantly higher in HER2 (p = 1.13E−05) and luminal-B (p = 6.67E−08) tumors compared to basal tumors. However, no difference in expression was observed between the luminal-B and the HER2 subtype (p = 4.39E−01). Higher expression was significantly associated with small tumor size (≤2 cm), low histological grade, and positive estrogen receptor status (Table 1).

**Table 1:** Characteristics of the untreated cohort and correlation with SPARC expression.

	N (%)	P-value	Direction
Total Number	948		
Age-≤50 years->50 years	474 (50%)474 (50%)	5.00E-02	Higher in older patients
Tumor size-≤2 cm->2 cm	617 (65.1%)331 (34.9%)	6.68E-07	Higher in smaller tumors
Nodal status-Node-negative-Node-positive	888 (93.7%)60 (6.3%)	8.60E-01	-
Histological grade-I-II-III	145 (15.3%)399 (42.1%)404 (42.6%)	1.34E-12	Higher in low grade tumors
Estrogen receptor status*-Negative-Positive	236 (25%)707 (75%)	7.64E-18	Higher in ER-positive tumors
Breast Cancer Subtype-Basal-HER2+-Luminal A-Luminal B	153 (16.1%)67 (7.1%)342 (36.1%)386 (40.7%)	1.57E-41	Higher in luminal-A tumors

* 5 patients had missing estrogen receptor status.

### Correlation between SPARC and Different Gene Modules

**Figure 2:**
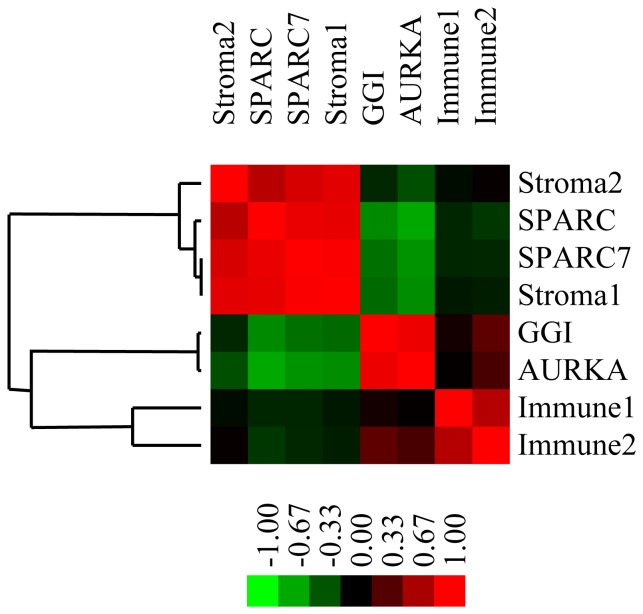
Heat map showing correlation between SPARC, SPARC7 and different gene modules in the systemically untreated cohort (n = 948). Red indicates positive and green indicates negative correlation.

We investigated whether SPARC and SPARC7 correlate with gene modules related to proliferation, immune and stroma (Figure 2). We found positive correlation between SPARC, and stroma-related gene modules; both stroma1 (r = 0.90) and stroma2 (r = 0.72, p = 1.31E−146). Among the 53 genes in SPARC7 module, 27 and 5 genes were also found in the stroma1 and stroma2 gene modules, respectively. The list of common genes between SPARC7 and the stroma-related modules are summarized in table S4. Five genes were common between SPARC7, stroma1 and stroma2 gene modules, which were ADAM metallopeptidase domain 12 (ADAM12), collagen type III, alpha1 (COL3A1), fibroblast activation protein (FAP), platelet-derived growth factor receptor beta (PDGFRB) and collagen type V alpha 2 (COL5A2).

On the other hand, we observed negative correlation between SPARC and proliferation-related gene modules; GGI (r = −0.55, p = 7.65E−75) and AURKA (r = −0.44, p = 3.17E−46). Low correlation was observed between SPARC or SPARC7 and immune-related gene modules.

Using the KEGG Pathway Database, we performed an exploratory analysis to evaluate pathways enriched according to SPARC expression. Detailed analyses are summarized in Table S5 and Table S6. At a false discovery rate (FDR) of <10%, we found 3 and 2 unique pathways enriched in SPARC low and high tumors, respectively. In tumors with low SPARC expression, there was up-regulation of the Mismatch repair pathway (p = 0.01, FDR = 0.08), the homologous recombination repair pathway (p = 0.004, FDR = 0.08) and DNA replication pathway (p = 0.01, FDR = 0.09). In tumors with high SPARC expression, there was up-regulation of the extracellular matrix receptor interaction pathway (p<0.0001, FDR = 0.03) and the focal adhesion pathway (p<0.0001, FDR = 0.04).

### Effect of SPARC Expression on Prognosis

To address the impact of SPARC on prognosis, we compared the risk of relapse among 3 groups of patients corresponding to the quantiles 33% and 66% of the expression of SPARC in each of the untreated dataset.

**Figure 3:**
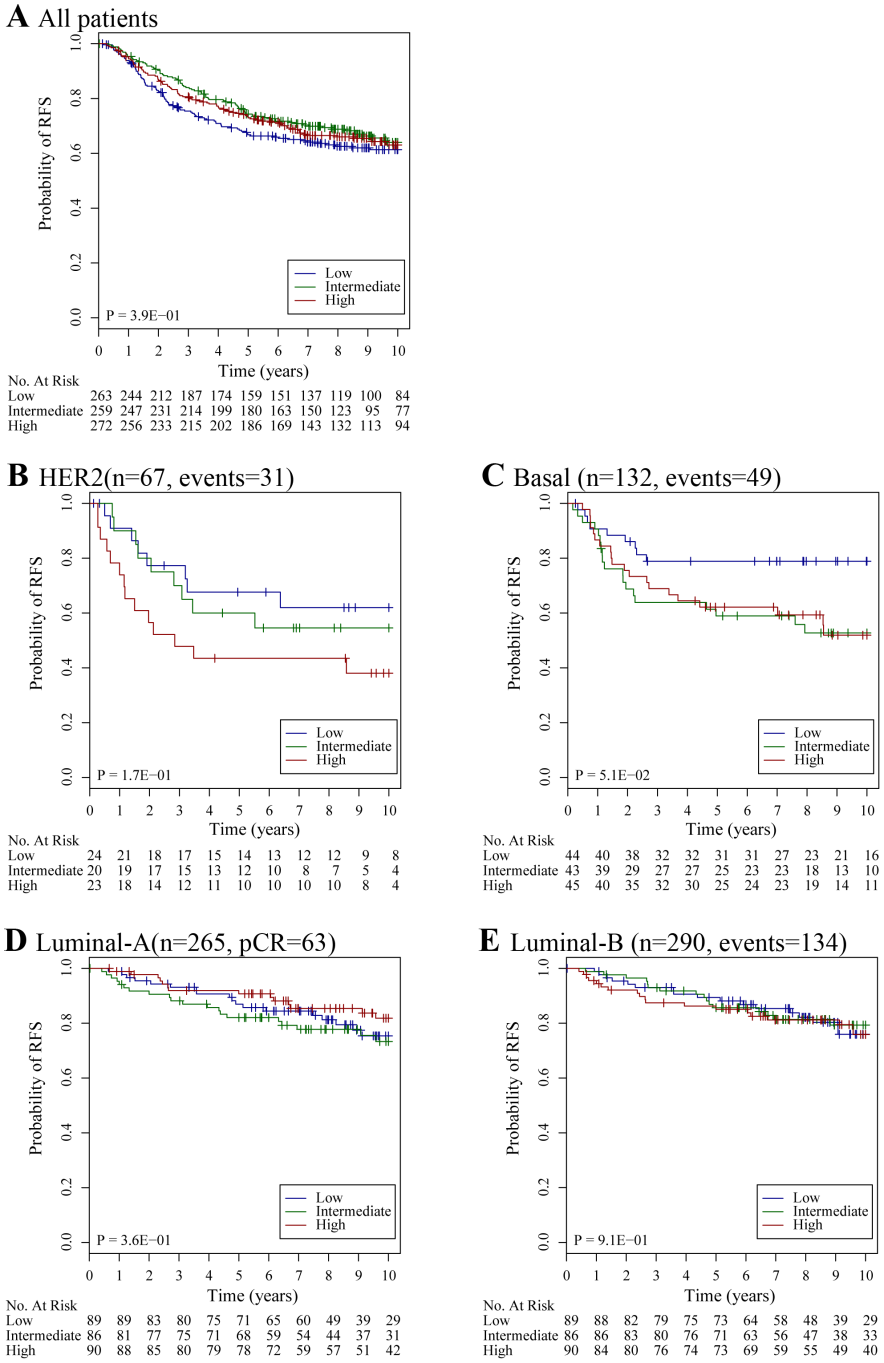
Association between SPARC expression and relapse free survival (RFS) in the systemically untreated cohort. **A)** All patients (n = 754, low: 251, intermediate: 243, high: 260); **B)** Patients with HER2 subtype (n = 67, low: 24, intermediate: 20, high: 23); **C)** Patients with basal subtype (n = 132, low: 44, intermediate: 43, high: 45); **D)** Patients with luminal-A subtype (n = 265; low: 89; intermediate: 86; high: 90) and luminal-B subtype (n = 290, low: 97, intermediate: 94, high: 99).

Information on RFS was missing in 194 patients (20.5%) and hence a total of 754 patients were eligible for this analysis. Median follow-up was 10 years (interquartile range: 7.7–12.4 years). The overall event rate was 36.7% (n = 277). On considering all patients, no difference in RFS was observed between the three groups (Figure 3A). However, on restricting the analysis according to breast cancer subtype, we found that high SPARC expression was associated with a trend of worse RFS in patients with basal and HER2-breast cancer (Figure 3B, 3C). No association was observed between SPARC expression and prognosis in the luminal subtypes (Figure 3D, 3E). The same results were observed with SPARC7 (Figure S1).

Table 2 summarizes the results of the multivariate model according to the breast cancer subtype for both SPARC and SPARC7. In the overall population, we found significant interaction between SPARC and ER status (p = 1.71E−02). Borderline interaction was observed between SPARC and HER2 (p = 9.33E−02). Same results were observed on replacing SPARC with SPARC7 (p = 1.63E−02) and (p = 1.59E−01) for ER and HER2 respectively. Subgroup analysis showed that patients with either basal or HER2 subtype and high SPARC expression had a higher risk of relapse even after adjusting for tumor size, nodal status, histological grade and age. This was not the case in patients diagnosed with luminal-A or luminal-B tumors.

**Table 2:** Multivariate model evaluating the association between SPARC, SPARC7 and relapse free survival in all patients and according to breast cancer subtype.

	Factors	HR [95% CI]	P-value
All patients	SPARCSPARC7*Tumor size (>2 cm vs. ≤2 cm)Nodal Status (positive vs. negative)Grade (III vs. II vs. I)Age (>50 vs.≤50)ER (positive vs. negative)HER2 (positive vs. negative)SPARC*ER (interaction)SPARC*HER2 (interaction)	1.2 [0.94–1.5]1.2 [0.99–1.6]1.3 [1.0–1.8]2.0 [1.2–3.2]1.4 [1.1–1.7]0.8 [0.6–1.1]0.9 [0.7–1.3]1.2 [0.8–1.8]0.5 [0.3–0.9]2.3 [0.9–6.2]	1.29E-015.32E-023.23E-023.69E-036.64E-041.86E-016.92E-013.14E-011.71E-029.33E-02
Basal	SPARCSPARC7 *Tumor size (>2 cm vs. ≤2 cm)Nodal Status (positive vs. negative)Grade (III vs. II vs. I)Age (>50 vs.≤50)	2.0 [1.1–3.7]2.2 [1.1–4.3]1.7 [0.8–3.9]0.5 [0.1–5.1]1.0 [0.48–2.1]0.6 [0.3–1.4]	2.28E-021.23E-021.77E-016.34E-019.98E-012.82E-01
HER2	SPARCSPARC7 *Tumor size (>2 cm vs. ≤2 cm)Nodal Status (positive vs. negative)Grade (III vs. II vs. I)Age (>50 vs.≤50)	2.3 [0.9–5.9]2.6 [1.1–6.7]1.6 [0.7–3.8]2.2 [0.8–6.3]0.9 [0.4–1.7]0.7 [0.8–0.3]	6.17E-023.80E-022.20E-011.30E-017.15E-017.36E-01
Luminal-A	SPARCSPARC7 *Tumor size (>2 cm vs. ≤2 cm)Nodal Status (positive vs. negative)Grade (III vs. II vs. I)Age (>50 vs.≤50)	0.8 [0.4–1.5]0.8 [0.4–1.6]1.8 [1.0–3.2]1.2 [0.4–3.7]1.3 [0.9–2.0]1.3 [0.8–2.3]	5.67E-016.14E-014.24E-027.20E-011.44E-012.81E-01
Luminal-B	SPARCSPARC7 *Tumor size (>2 cm vs. ≤2 cm)Nodal Status (positive vs. negative)Grade (III vs. II vs. I)Age (>50 vs.≤50)	1.2 [0.8–1.7]1.3 [0.9–1.8]1.1 [0.7–1.6]2.1 [1.0–4.4]1.3 [1.0–1.8]0.7 [0.8–0.3]	3.67E-011.56E-016.16E-013.48E-012.87E-021.28E-01

* on inserting SPARC7 instead of SPARC in the model.

### Effect of SPARC Expression on Response to Neoadjuvant Chemotherapy

**Figure 4:**
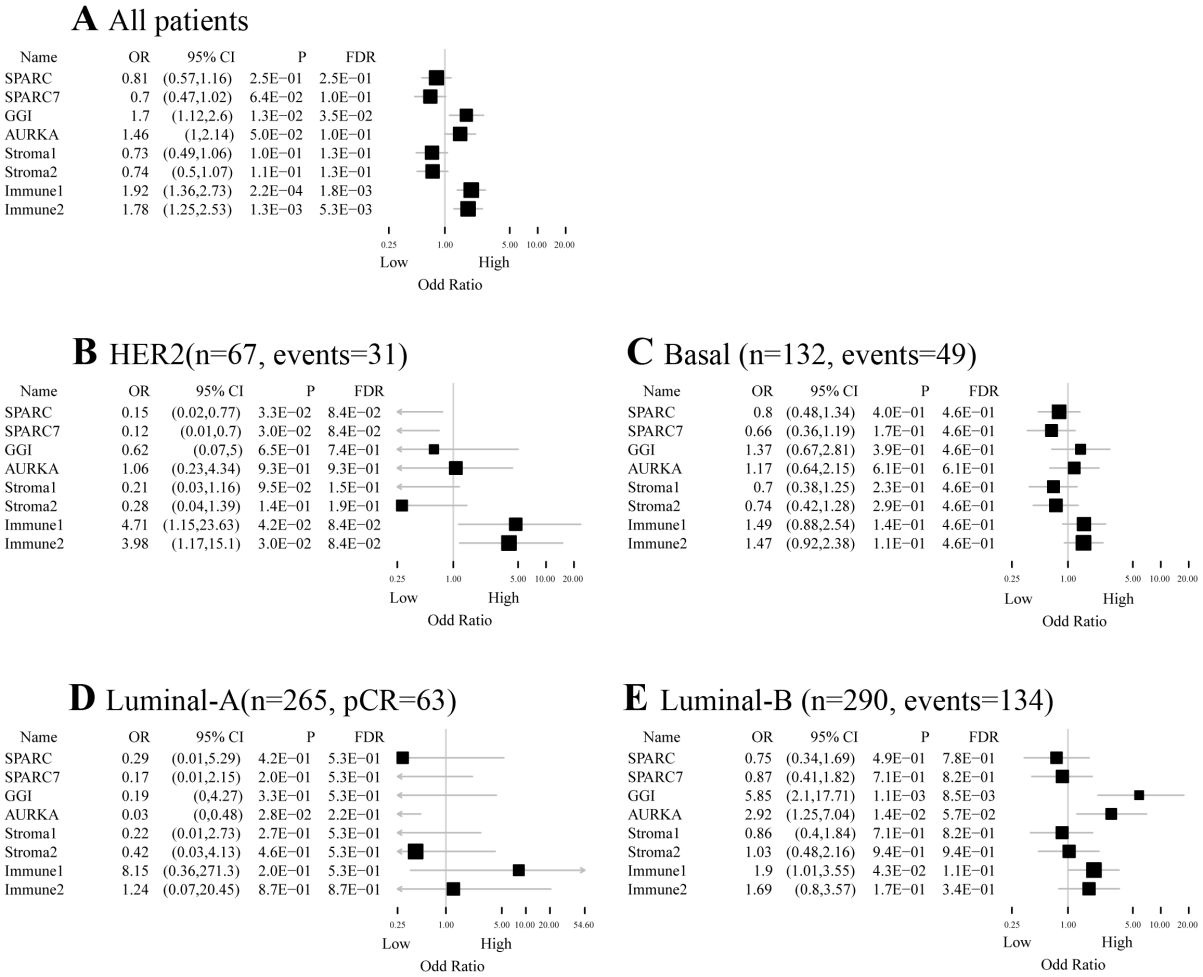
ORs for pCR for unit increase in module score after adjustment in a logistic regression model. **A)** All patients (n = 791, pCR = 175); **B)** Patients with HER2 subtype (n = 86, pCR = 22); **C)** Patients with basal subtype (n = 316, pCR = 106); **D)** Patients with luminal-A subtype (n = 129, pCR = 6); **E)** Patients with luminal-B subtype (n = 260, pCR = 41). FDR, false discovery rate.

We evaluated whether SPARC or SPARC7 is associated with response to neoadjuvant chemotherapy. Out of 791 eligible patients, 175 had a pCR (22%). In a multivariate model, we did not find a significant association between SPARC or SPARC7 and pCR (Figure 4A). No significant interaction was observed between SPARC and ER (p = 1.85E−01) or HER2 (p = 5.21E−01). For SPARC7, there was a significant interaction with ER (p = 3.69E−02) but not HER2 (p = 1.17–01). A subgroup analysis according to breast cancer subtype showed that high SPARC expression was independently associated with low pCR rate, only in the HER2 subtype (OR: 0.15; 95% CI [0.02–0.77], p = 3.3E−02) (Figure 4B). This was not observed in patients with basal or luminal breast cancer (Figure 4C–E).

## Discussion

To our knowledge, this is the largest analysis that addresses the association between SPARC expression, prognosis and response to neoadjuvant chemotherapy in early breast cancer. We found that SPARC is highly expressed in ER-positive, small and slowly proliferating tumors. SPARC expression was associated with prognosis in patients with basal-like tumors. However, less obvious associations were observed regarding response to treatment. Of note, none of the previous reports have investigated the expression or effect of SPARC expression on outcome according to molecular subtypes.

We observed high association with stroma-related gene modules, which is in line with the role of SPARC in regulating the ECM. GSEA analysis further showed up-regulation of the extracellular matrix pathway in tumors with high SPARC expression confirming this association. SPARC has been shown to inhibit cell cycle progression, which is reflected in our analysis by its negative correlation with proliferation-related gene modules. This is also concordant with the association observed with clinical parameters. High SPARC expression was observed with low histological grade, small tumor size, and high ER expression, which are all features of slowly proliferating tumors. On the other hand, low SPARC expression was observed in basal tumors and was associated with up-regulation of DNA repair pathways, which are also known to be altered in basal tumors [29]. While our analyses are consistent with each other and the known functions of SPARC, results of previous studies were not as constant in this regard. Helleman et al [17] have reported that high SPARC expression by RT-PCR is associated with high grade, positive nodal status, but small tumor size while a recent study [19] has shown that high SPARC expression by immunohistochemistry is associated with small tumor size, but high nuclear grade and HER2 expression.

We found that high SPARC mRNA expression was associated with poor prognosis in patients diagnosed with basal and HER2-positive breast cancer. This is in contrast with recent data from Nagai et al [19], showing that low SPARC expression using IHC is associated with poor prognosis. However, it is important to acknowledge that different technologies were used to evaluate SPARC across the different studies. In our analysis, we evaluated the expression of SPARC at the mRNA level as a continuum, which reduces the potential bias that could be associated with the use of cut-offs to assign SPARC positivity at the protein level which has been adopted by the other groups.

Recently, we reported the association between different molecular processes and response to primary chemotherapy [20]. We found significant association between high immune response and high rates of pCR in patients with HER2-positive breast cancer. However, no significant association with stroma-related gene modules was found. In the current analysis, we found a significant association between high SPARC expression and low pCR rates in the HER2 subtype, using the same dataset. However, we failed to show a significant interaction between HER2 and SPARC expression in the overall population. Hence, caution should be made in interpreting this data, which would require further confirmation. Previously, Desai et al have suggested that in HER2-positive tumors, SPARC expression may play a role in determining response to taxanes [30]. In their experiment, they found that albumin-bound paclitaxel was more effective than conventional paclitaxel in HER2-positive cell lines, but only in the presence of high SPARC expression. This was running in line with prior reports suggesting that SPARC improves intra-tumor concentration of paclitaxel when an albumin-bound preparation is used given the high affinity of SPARC to albumin [31]. In the current study, patients were treated in the neoadjuvant setting with anthracyclines with or without taxanes, but none were treated with albumin-bound paclitaxel. The latter is currently approved in the treatment of metastatic breast cancer [32]; however, we lack any biomarker that could identify patients that may benefit this drug. Preliminary results in head and neck and pancreatic cancer have suggested that high SPARC expression could be also associated with response to albumin-bound paclitaxel [33], [34]. Acknowledging these facts and based on our results, it would be interesting to validate the association between SPARC expression and the benefit of albumin-bound cytotoxics in HER2-positive breast cancer.

In conclusion, the current analysis suggests a potential role of SPARC in determining prognosis and response to primary chemotherapy in early breast cancer. This information could guide further development of albumin-bound cytotoxics in breast cancer.

## Supporting Information

Figure S1 **The association between SPARC7 expression and relapse free survival (RFS) in the systemically untreated cohort.** A) All patients (n = 754, low: 251, intermediate: 243, high: 260); **B)** Patients with HER2 subtype (n = 67, low: 24, intermediate: 20, high: 23); **C)** Patients with basal subtype (n = 132, low: 44, intermediate: 43, high: 45); **D)** Patients with luminal-A subtype (n = 265; low: 89; intermediate: 86; high: 90) and luminal-B subtype (n = 290, low: 97, intermediate: 94, high: 99).
(PPTX)

Table S1 **The list of genes in the different gene modules.**
(XLSX)

Table S2 **List of eligible datasets included in the systemically untreated cohort (10 datasets, 948 patients).**
(DOCX)

Table S3 **SPARC7 gene list.**
(DOCX)

Table S4 **List of common genes between SPARC7 and stroma-related modules. A)** SPARC7– Stroma1 (DCN), n = 27; **B)** SPARC7– Stroma2 (PLAU), n = 5.
(DOCX)

Table S5 **GSEA analysis showing pathways that are upregulated in tumors with low SPARC expression (p-value and FDR value are provided).**
(XLSX)

Table S6 **GSEA analysis showing pathways that are upregulated in tumors with high SPARC expression (p-value and FDR value are provided).**
(XLSX)
