# Correction: Association between SPARC mRNA Expression, Prognosis and Response to Neoadjuvant Chemotherapy in Early Breast Cancer: A Pooled *in-silico* Analysis

**DOI:** 10.1371/annotation/3d5a5933-791f-4191-98f5-f559a872e404

**Published:** 2013-12-16

**Authors:** Hatem A. Azim, Sandeep Singhal, Michail Ignatiadis, Christine Desmedt, Debora Fumagalli, Isabelle Veys, Denis Larsimont, Martine Piccart, Stefan Michiels, Christos Sotiriou

There are multiple errors in the titles for Figure 3 and 4. The correct titles for the figures are:

In figure 3D: the tile should be: Luminal A (n=265, events=63)

In figure 4A: the title should be: All patients (n=791, pCR=175)

In figure 4B: the title should be HER2 (n=86, pCR=22)

In figure 4C: the title should be Basal (n=316, pCR=106)

In figure 4D: the title should be Luminal-A (n=129, pCR=6)

In figure 4E: the title should be Luminal-B (n=260, pCR=41)

