# Supplementary material for: Association between SPARC mRNA Expression, Prognosis and Response to Neoadjuvant Chemotherapy in Early Breast Cancer: A Pooled in-silico Analysis
Source: PLoS One. 2013 Apr 26;8(4):e62451. doi: 10.1371/journal.pone.0062451 (PMC3637211; doi:10.1371/journal.pone.0062451)
Supplement: Table S2 — List of eligible datasets included in the systemically untreated cohort (10 datasets, 948 patients). (DOCX) [file pone.0062451.s003.docx]

**Table S2:** List of datasets included in the systemically untreated cohort

| Dataset | Number of patients (%) | References |
| --- | --- | --- |
| CAL | 13 (1.4%) | [1] |
| MAINZ | 194 (20.5%) | [2] |
| NCI | 10 (1.1%) | [3] |
| NKI | 189 (19.9%) | [4,5] |
| STNO2 | 16 (1.7) | [6] |
| TRANSBIG | 187 (19.7%) | [7] |
| UNT | 58 (6.1%) | [8] |
| UPP | 86 (9.1%) | [9] |
| VDX | 195 (20.6%) | [10] |
| Total | 948 |  |

**References**

1. Chin K, DeVries S, Fridlyand J, Spellman PT, Roydasgupta R, et al. (2006) Genomic and transcriptional aberrations linked to breast cancer pathophysiologies. Cancer Cell 10: 529-541.

2. Schmidt M, Bohm D, von Torne C, Steiner E, Puhl A, et al. (2008) The humoral immune system has a key prognostic impact in node-negative breast cancer. Cancer Res 68: 5405-5413.

3. Sotiriou C, Neo SY, McShane LM, Korn EL, Long PM, et al. (2003) Breast cancer classification and prognosis based on gene expression profiles from a population-based study. Proc Natl Acad Sci U S A 100: 10393-10398.

4. van 't Veer LJ, Dai H, van de Vijver MJ, He YD, Hart AA, et al. (2002) Gene expression profiling predicts clinical outcome of breast cancer. Nature 415: 530-536.

5. van de Vijver MJ, He YD, van't Veer LJ, Dai H, Hart AA, et al. (2002) A gene-expression signature as a predictor of survival in breast cancer. N Engl J Med 347: 1999-2009.

6. Sorlie T, Tibshirani R, Parker J, Hastie T, Marron JS, et al. (2003) Repeated observation of breast tumor subtypes in independent gene expression data sets. Proc Natl Acad Sci U S A 100: 8418-8423.

7. Desmedt C, Piette F, Loi S, Wang Y, Lallemand F, et al. (2007) Strong time dependence of the 76-gene prognostic signature for node-negative breast cancer patients in the TRANSBIG multicenter independent validation series. Clin Cancer Res 13: 3207-3214.

8. Sotiriou C, Wirapati P, Loi S, Harris A, Fox S, et al. (2006) Gene expression profiling in breast cancer: understanding the molecular basis of histologic grade to improve prognosis. J Natl Cancer Inst 98: 262-272.

9. Miller LD, Smeds J, George J, Vega VB, Vergara L, et al. (2005) An expression signature for p53 status in human breast cancer predicts mutation status, transcriptional effects, and patient survival. Proc Natl Acad Sci U S A 102: 13550-13555.

10. Wang Y, Klijn JG, Zhang Y, Sieuwerts AM, Look MP, et al. (2005) Gene-expression profiles to predict distant metastasis of lymph-node-negative primary breast cancer. Lancet 365: 671-679.
