# Supplementary material for: Association between SPARC mRNA Expression, Prognosis and Response to Neoadjuvant Chemotherapy in Early Breast Cancer: A Pooled in-silico Analysis
Source: PLoS One. 2013 Apr 26;8(4):e62451. doi: 10.1371/journal.pone.0062451 (PMC3637211; doi:10.1371/journal.pone.0062451)
Supplement: Table S3 — SPARC7 gene list. (DOCX) [file pone.0062451.s004.docx]

**Table S3:** The SPARC7 module gene list and their correlation with SPARC

| **Probe** | **Correlation with the SPARC gene (coefficient)** |
| --- | --- |
| SPARC | 1 |
| COL1A2 | 0.884496 |
| HTRA1 | 0.87898 |
| COL3A1 | 0.846678 |
| COL5A2 | 0.842419 |
| C1QTNF6 | 0.827824 |
| COL6A3 | 0.822736 |
| SFRP2 | 0.820405 |
| COL12A1 | 0.819032 |
| ASPN | 0.813438 |
| GLT8D2 | 0.811735 |
| PPAPDC1A | 0.803854 |
| THBS2 | 0.801989 |
| DACT1 | 0.783263 |
| AEBP1 | 0.779848 |
| CRISPLD2 | 0.779284 |
| P4HA3 | 0.773135 |
| MMP2 | 0.770026 |
| COPZ2 | 0.769066 |
| MSRB3 | 0.764456 |
| ADAM12 | 0.763295 |
| LOXL1 | 0.762087 |
| NID2 | 0.760073 |
| EFEMP2 | 0.756635 |
| CTHRC1 | 0.753395 |
| SPON1 | 0.751128 |
| FAP | 0.748348 |
| CTSK | 0.744412 |
| PDGFRL | 0.743383 |
| NDN | 0.742497 |
| TSHZ3 | 0.741341 |
| LRRC15 | 0.741332 |
| C1QTNF3 | 0.739576 |
| NUAK1 | 0.738229 |
| C20orf39 | 0.737629 |
| FSTL1 | 0.737569 |
| MXRA5 | 0.73694 |
| COL1A1 | 0.734077 |
| FIBIN | 0.732603 |
| POSTN | 0.732565 |
| ITGBL1 | 0.731798 |
| PDGFRB | 0.731387 |
| PODN | 0.730073 |
| COL10A1 | 0.721348 |
| C16orf30 | 0.718408 |
| CMTM3 | 0.711768 |
| SRPX2 | 0.711204 |
| DKK3 | 0.710686 |
| PCOLCE | 0.710575 |
| SERPINF1 | 0.706977 |
| LAMB1 | 0.704486 |
| C10orf56 | 0.703521 |
| ECM2 | 0.700331 |
