# Supplementary material for: Association between SPARC mRNA Expression, Prognosis and Response to Neoadjuvant Chemotherapy in Early Breast Cancer: A Pooled in-silico Analysis
Source: PLoS One. 2013 Apr 26;8(4):e62451. doi: 10.1371/journal.pone.0062451 (PMC3637211; doi:10.1371/journal.pone.0062451)
Supplement: Table S4 — List of common genes between SPARC7 and stroma-related modules. A) SPARC7– Stroma1 (DCN), n = 27; B) SPARC7– Stroma2 (PLAU), n = 5. (DOCX) [file pone.0062451.s005.docx]

**Table S4:** The list of common genes between SPARC7 and stroma-related modules

1. SPARC7 – stroma1 (DCN) ; n=27

| **Gene Symbol** | **EntrezGene.ID** |
| --- | --- |
| SPARC | 6678 |
| COL1A2 | 1278 |
| HTRA1 | 5654 |
| COL3A1 | 1281 |
| COL5A2 | 1290 |
| COL6A3 | 1293 |
| ASPN | 54829 |
| THBS2 | 7058 |
| DACT1 | 51339 |
| AEBP1 | 165 |
| MMP2 | 4313 |
| COPZ2 | 51226 |
| ADAM12 | 8038 |
| LOXL1 | 4016 |
| SPON1 | 10418 |
| FAP | 2191 |
| CTSK | 1513 |
| PDGFRL | 5157 |
| NDN | 4692 |
| C1QTNF3 | 114899 |
| POSTN | 10631 |
| ITGBL1 | 9358 |
| PDGFRB | 5159 |
| COL10A1 | 1300 |
| PCOLCE | 5118 |
| SERPINF1 | 5176 |
| ECM2 | 1842 |

1. SPARC7 – stroma2 (PLAU); n=5

| **Gene Symbol** | **EntrezGene.ID** |
| --- | --- |
| ADAM12 | 8038 |
| COL3A1 | 1281 |
| FAP | 2191 |
| PDGFRB | 5159 |
| COL5A2 | 1290 |
